# Tumour Localisation With ICG‐Soaked Embolisation Coils Without Robotic Bronchoscopy: First German Experience

**DOI:** 10.1002/rcr2.70491

**Published:** 2026-01-30

**Authors:** Sammy Onyancha, Hasnain Bawaadam, Ramin Lonnes, Peter Hollaus, Kati Kiil, Gernot Rohde, Waldemar Schreiner

**Affiliations:** ^1^ Department of Pulmonology St. Elisabethen Krankenhaus Frankfurt Germany; ^2^ Department of Pulmonary and Critical Care Medicine Aurora Medical Center Kenosha Kenosha Wisconsin USA; ^3^ Department of Thoracic Surgery St. Elisabethen Krankenhaus Frankfurt Germany; ^4^ Dr. Senckenbergisches Institut für Pathologie Frankfurt am Main Germany; ^5^ Department of Respiratory Medicine Universitätsklinikum Marburg Marburg Germany

**Keywords:** fluorescence, ICG, indocyanine green, lung sparring sublobar resection, VATS

## Abstract

Accurate localisation of small or non‐palpable pulmonary lesions is critical for successful minimally invasive resection. Indocyanine green (ICG)‐soaked embolisation coil placement is an emerging fluorescence‐guided marking method, typically applied to subpleural nodules. We present a four‐patient case series—the first in Germany—where ICG‐soaked embolisation coils were placed using ultrathin bronchoscopy, a standard cytology brush catheter and cone beam CT (CBCT) guidance, without robotic bronchoscopy. Lesions were confirmed with radial endobronchial ultrasound (EBUS), and resection was performed via uniportal video‐assisted thoracoscopic surgery (VATS). Marking was successful in all cases without complications. One patient underwent dual‐coil placement to enable three‐dimensional bracketing of a non‐subpleural lesion, facilitating precise anatomical resection. Histologies included squamous cell carcinoma (*n* = 1), adenocarcinoma (*n* = 1), hamartoma (*n* = 1) and typical carcinoid (*n* = 1). All patients achieved R0 resections. ICG‐soaked coil placement via ultrathin bronchoscopy is a safe, reproducible and effective localisation technique, even in centres without robotic navigation systems. This series demonstrates feasibility across diverse lesion locations and expands the applicability of fluorescence‐guided thoracic surgery to more centrally oriented nodules.

## Graphical abstract

Accurate localisation of small or non‐palpable pulmonary lesions is critical for successful minimally invasive resection. We present a four‐patient case series—the first in Germany—where ICG‐soaked embolisation coils were placed using ultrathin bronchoscopy, a standard cytology brush catheter and cone beam CT (CBCT) guidance, without robotic bronchoscopy.

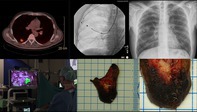

## 1 Introduction

The rising detection rate of small pulmonary nodules through low dose computed tomography (CT) screening and advanced imaging has increased the demand for precise intraoperative localisation techniques. In minimally invasive thoracic surgery, especially uniportal video‐assisted thoracoscopic surgery (VATS), the absence of tactile feedback poses a challenge for identifying such lesions, particularly when they are small, deep, or non‐subpleural.

Preoperative bronchoscopic localisation techniques have evolved to meet this need [1, 2, 3, 4, 5]. Among these, the deployment of embolisation coils soaked in indocyanine green (ICG) has gained attention due to its dual benefits: the coil provides a radiopaque marker visible under fluoroscopy, while the ICG offers strong fluorescence for near‐infrared (NIR) visualisation during surgery [6]. This combination enables real‐time intraoperative guidance and facilitates precise, lung‐sparing resections.

Most published experiences with ICG coil marking involve robotic bronchoscopy or electromagnetic navigation bronchoscopy (ENB) systems [6, 7, 8], which can limit adoption in hospitals without such technology. Furthermore, conventional single‐coil placement is optimised for subpleural nodules and may be less effective for centrally oriented lesions, where tissue depth reduces fluorescence signal.

In this context, we report the first German case series of patients undergoing ICG‐soaked embolisation coil placement using only ultrathin bronchoscopy, a standard cytology brush catheter and cone beam CT (CBCT) guidance without robotic assistance. This approach underscores the reproducibility and broad accessibility of the technique. Additionally, one case demonstrates a novel dual‐coil ‘bracketing’ strategy for precise three‐dimensional localisation of a non‐subpleural nodule.

## 2 Case Series

Four patients with peripheral pulmonary nodules underwent bronchoscopic localisation using ICG‐soaked embolisation coils. Patient characteristics, lesion features, procedural details and operative outcomes are summarised in Table 1. All four procedures were performed under total intravenous anaesthesia with jet ventilation. Cases 1, 2 and 4 were carried out as flexible bronchoscopies through an endotracheal tube with a jet‐converter, whereas Case 3 was performed using rigid bronchoscopy (Video 1). ICG was prepared at a concentration of 5 mg/mL. In this series, endobronchial ICG injection without coils was not performed. Previous studies have shown that instillation of ICG into segmental bronchi can delineate intersegmental planes, but diffusion of dye limits delayed resection and can reduce precision for small or deep nodules. In contrast, ICG‐soaked coils provide both a durable radiopaque marker and localised fluorescence. Furthermore, they minimise diffusion, thus improving intraoperative accuracy and allowing delayed resection.

**TABLE 1:** Summary of cases—patient characteristics and procedural data.

Case	Age	Localisation	No. of coils	Coil to pleura distance	Bronchoscopy time	Complication	Days between implantation and resection	Operation time	R0 resection	Histology
Case 1	57	RLL/S6	1	12 mm	42 min	None	7 days	190 min	Yes	Squamous cell carcinoma
Case 2	45	RUL/S3	2	Proximal coil: 26 mm Distal coil: 8 mm	55 min	None	4 days	135 min	Yes	Typical carcinoid
Case 3	49	RUL/S1	1	10 mm	49 min	None	2 days	118 min	Yes	Adenocarcinoma
Case 4	59	LUL/S4	1	7 mm	45 min	None	2 days	203 min	Yes	Hamartoma

**VIDEO 1:** Bronchoscopic implantation of ICG‐dyed coil as well as NIR‐imaging visualisation of the coil during uniportal VATS. Video content can be viewed at https://onlinelibrary.wiley.com/doi/10.1002/rcr2.70491.

### 2.1 Case 1

**FIGURE 1:**
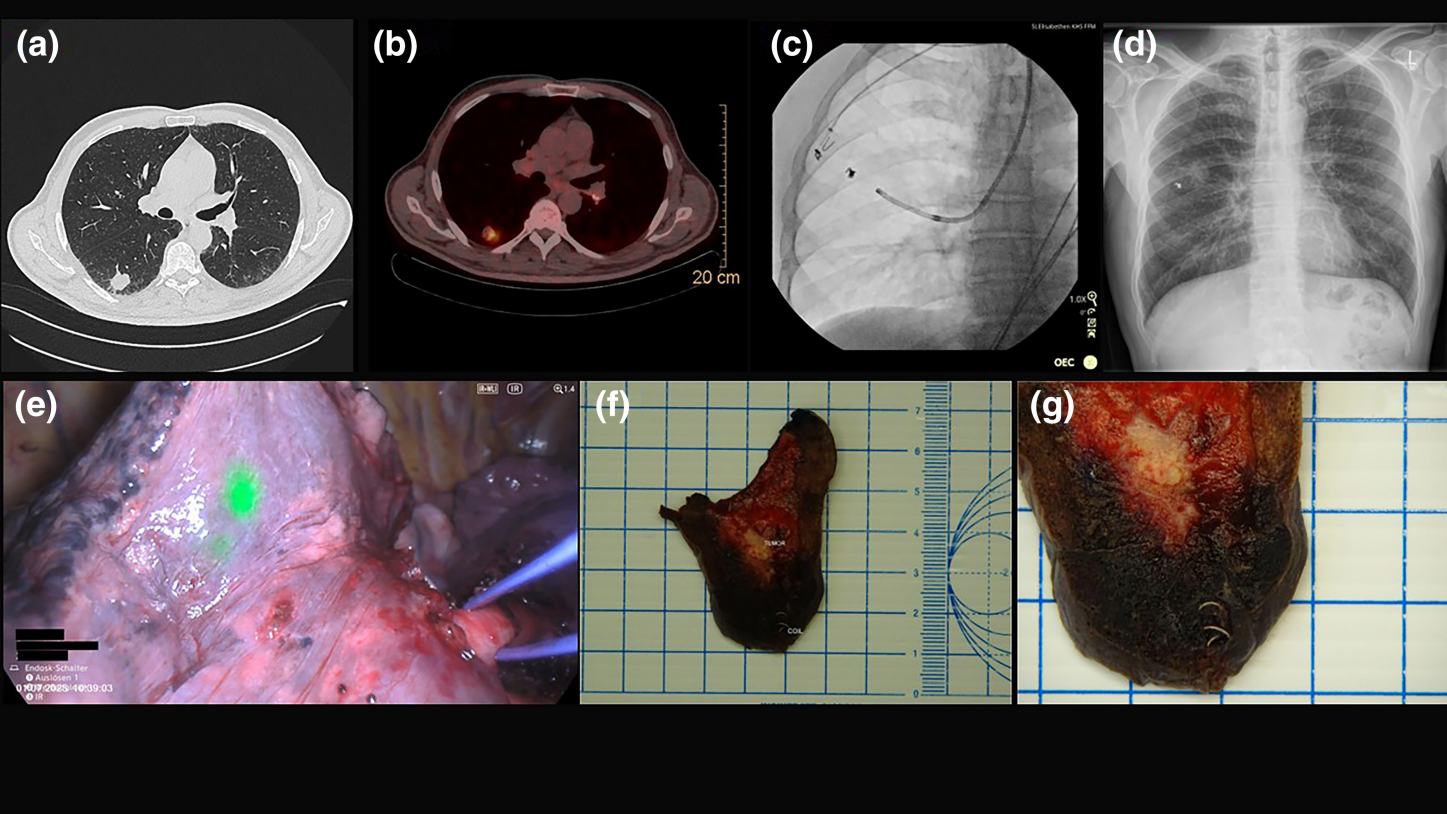
(a) CT scan showing lesion in right lower lobe. (b) PET‐CT scan showing avid FDG uptake in the lower lobe lesion (S6). (c) Coil‐placement under CBCT and fluoroscopy guidance. (d) Postinterventional chest x‐ray showing coil placement in right lower lobe. (e) NIR thoracoscopy showing ICG‐coil fluorescence. (f and g) Lung specimen showing coil placement < 1 cm away from tumour.

A 57‐year‐old male with a history of smoking presented with a solitary right lower lobe segment 6 lesion identified on follow‐up CT for prior pneumonia (Figure 1). The lesion measured 14 mm in diameter, was peripheral and had no pleural contact. PET‐CT showed avid FDG uptake SUV_max_ = 4.5 without nodal or extrapulmonary involvement (Figure 1b).

Ultrathin bronchoscopy (BF‐MP190F; Olympus Medical Systems, Japan) was used to navigate to the subsegmental bronchus under virtual bronchoscopy guidance. Radial EBUS confirmed a concentric lesion signal. One embolisation coil (MWCE‐35‐7/3 Tornado embolisation coil, 7 × 3 mm; Cook Medical, Germany) soaked in ICG for 10 min. Under CBCT guidance (OEC 3D, GE Healthcare, USA), the coil was deployed directly adjacent to the lesion using a 1.7 mm cytology brush (BC‐205D‐2010; Olympus Medical Systems, Japan) after the coil was retrogradely loaded into the brush's catheter using a guidewire (Figure 1c). Post‐deployment CBCT confirmed correct position, and no pneumothorax was observed (Figure 1d). The bronchoscopy, including coil deployment, required approximately 42 min.

Seven days later, the patient underwent uniportal VATS S6 segmentectomy. The coil was clearly visible under NIR thoracoscopic imaging (Figure 1e), guiding the resection plane. Postoperative recovery was uneventful, and the patient was discharged on Day 5. Histology confirmed squamous cell carcinoma with R0 resection (Figure 1f,g).

### 2.2 Case 2

**FIGURE 2:**
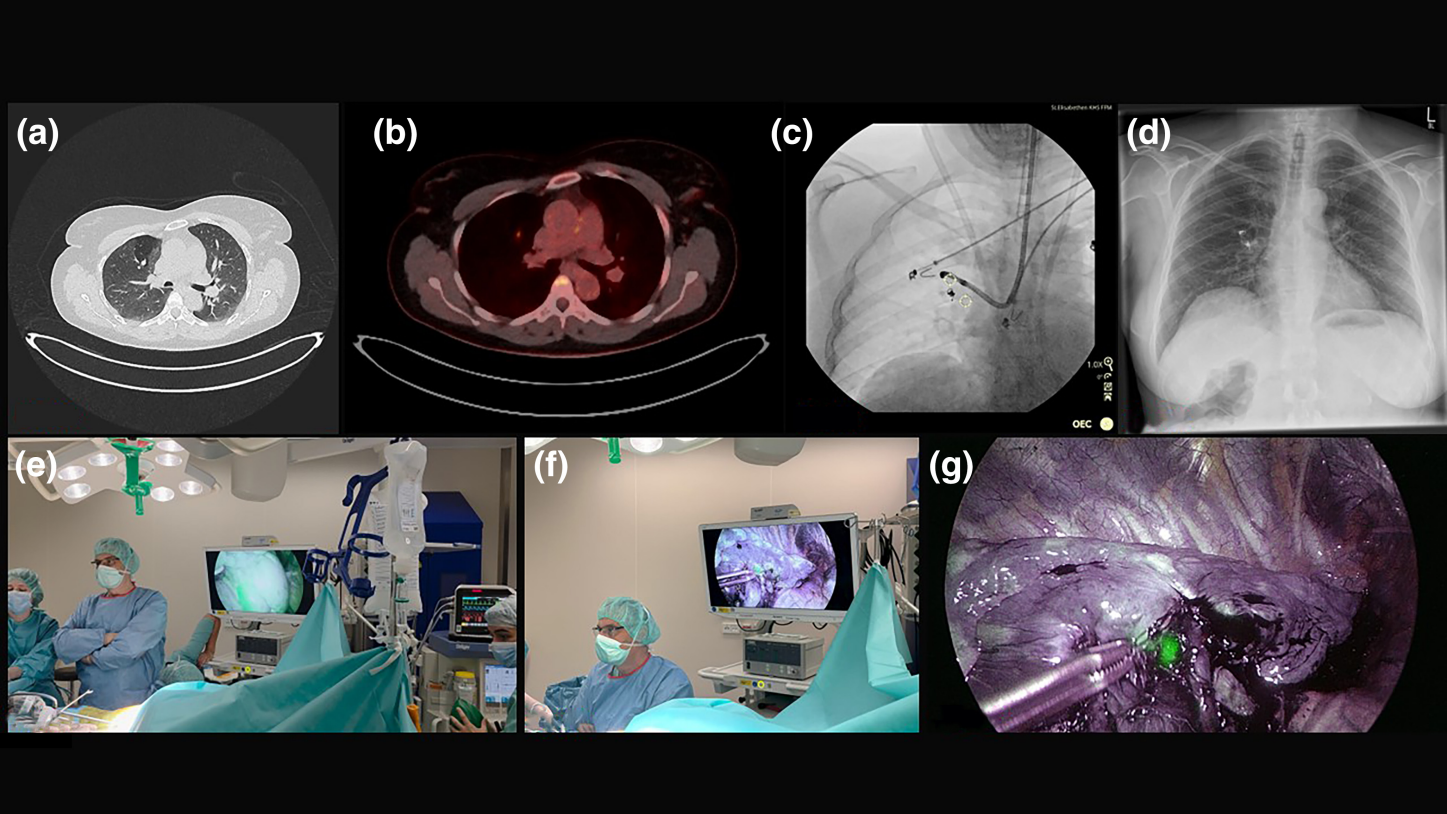
(a) CT scan showing lesion in right upper lobe (S3). (b) PET‐CT scan with FDG uptake in the right upper lobe lesion. (c) Dual coil‐placement in right upper lobe under fluoroscopic guidance. (d) Postinterventional chest x‐ray showing positioning of both coils in right upper lobe. (e) NIR thoracoscopy identification of first coil adjacent to the pleura. (f and g) NIR thoracoscopy identification of second coil adjacent to the tumour.

A 45‐year‐old female with no smoking history was found to have a PET‐avid 9 mm nodule in the anterior segment of the right upper lobe during workup for an unrelated trauma (Figure 2a). PET‐CT demonstrated SUV_max_ 6.8 uptake without mediastinal lymphadenopathy (Figure 2b). The nodule was peripheral in imaging appearance but lacked direct pleural contact, raising concern about intraoperative localisation.

An ultrathin bronchoscope was navigated to the target bronchus using virtual bronchoscopy planning. Radial EBUS confirmed proximity to the lesion. CBCT was performed to map anatomy precisely. Two Tornado embolisation coils (MWCE‐35‐6/3 Tornado embolisation coil, 6 × 3 mm; Cook Medical, Germany and MWCE‐35‐7/3 Tornado embolisation coil, 7 × 3 mm; Cook Medical, Germany), each soaked in ICG for 10 min, were deployed: the first distally at the pleural boundary of the segment via guidewire due to angulated access and the second proximally adjacent to the lesion via cytology brush catheter (Figure 2c). CBCT confirmed both coil positions, and no pneumothorax occurred (Figure 2d). The total procedural duration was 55 min.

Surgery took place 4 days later. Under NIR imaging, both coils were visible and created a three‐dimensional ‘bracketing’ effect (Figure 2f–h), enabling precise uniportal VATS S3 segmentectomy without conversion. Postoperative recovery was uneventful, and the patient was discharged on Day 4. Pathology confirmed typical carcinoid with R0 margins.

### 2.3 Case 3

**FIGURE 3:**
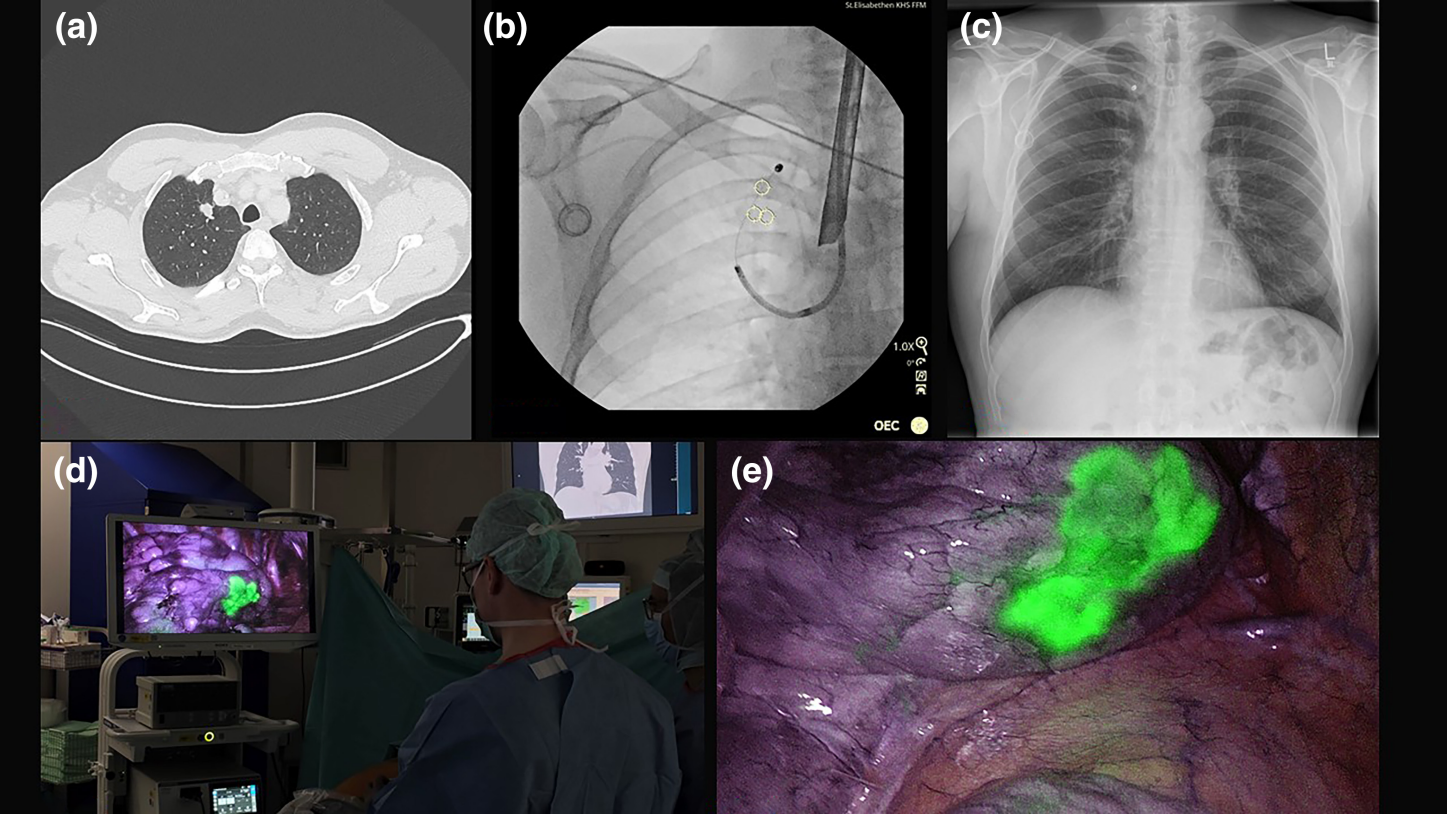
(a) CT scan showing lesion in right upper lobe (S1). (b) Fluoroscopy guided coil deployment in right upper lobe. (c) Postinterventional chest x‐ray showing no pneumothorax and presence of coil in right upper lobe. (d and e) NIR thoracoscopic imaging used to localise the tumour.

A 49‐year‐old male smoker with a history of colon cancer was referred for evaluation of a right upper lobe apical segment nodule detected on CT (Figure 3a). The 11 mm lesion was peripheral and demonstrated low‐grade PET uptake.

Bronchoscopic marking was performed using rigid bronchoscopy under total intravenous anaesthesia with jet ventilation (Twinstream, Carl Reiner GmbH, Austria). An ultrathin bronchoscope was navigated to the apical segment, and radial EBUS confirmed lesion location. CBCT was used for trajectory confirmation. A single ICG‐soaked coil was deployed using the 1.7 mm cytology brush with a 0.2 mL ICG flush via the brush's catheter post deployment to ensure pleural fluorescence (Figure 3b).

CBCT confirmed correct placement and pneumothorax was ruled out in the postinterventional chest x‐ray (Figure 3c). The total duration of the bronchoscopic procedure was 49 min.

The patient proceeded to uniportal VATS S1 segmentectomy 2 days later. The coil was readily identified under NIR imaging, guiding resection (Figure 3d,e). Pathology revealed adenocarcinoma.

### 2.4 Case 4

**FIGURE 4:**
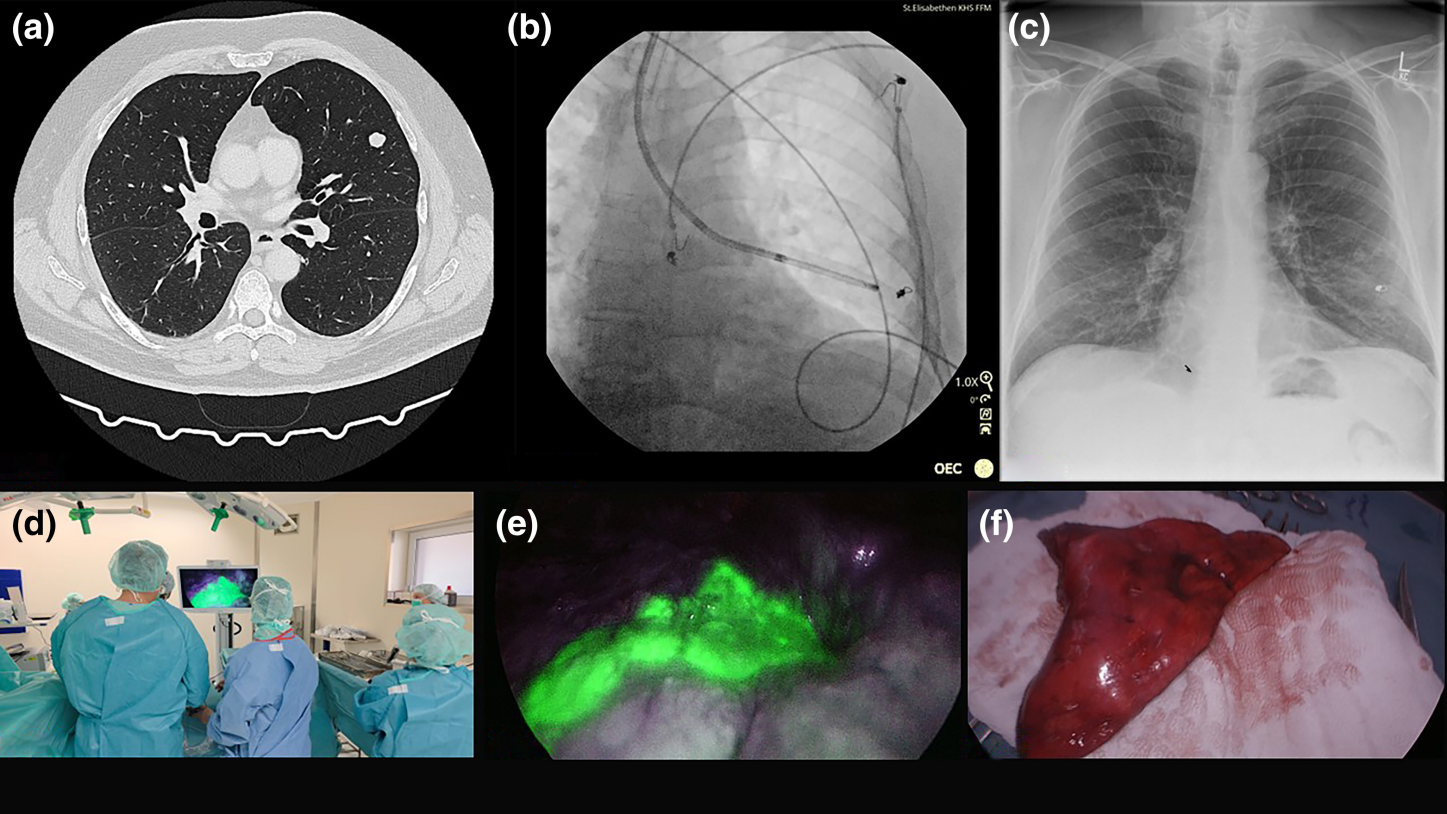
(a) CT scan showing lesion in left upper lobe (LB4). (b) Coil placement in lingula segment under fluoroscopy. (c) Postinterventional chest x‐ray showing coil placement in left upper lobe. (d and e) NIR thoracoscopic imaging visualisation of the tumour left upper lobe. (f) Lung specimen of S4 under white light.

A 59‐year‐old male, ex‐smoker, was found to have a 13 mm lesion in the lingula segment of the left upper lobe during evaluation for persistent cough (Figure 4a). Previous diagnostic bronchoscopy had ruled out lymph node involvement. PET‐CT showed moderate FDG uptake and confirmed the absence of nodal disease.

The ultrathin bronchoscope was advanced to LB4 under virtual bronchoscopy guidance (Archimedes, Broncus Medical, USA), with radial EBUS confirmation. CBCT imaging defined the lesion's relationship to the bronchial tree. A single ICG‐soaked coil was delivered using the BC‐205D cytology brush (Figure 4b). Coil position was verified by CBCT, and no complications occurred (Figure 4c). The total duration of the procedure was 45 min.

Surgery took place 2 days later via uniportal VATS where a S4 segmentectomy was performed. The fluorescent coil was visualised under NIR, enabling precise anatomical segment resection (Figure 4d–f). The patient's postoperative course was uneventful. Histology confirmed hamartoma with R0 margins.

## 3 Discussion

Accurate preoperative localisation of small pulmonary nodules remains a critical determinant of success in minimally invasive thoracic surgery. The shift towards lung‐sparing resections such as segmentectomies and wedge resections has made precision even more important, especially for nodules that are impalpable, deep‐seated, or not visible on the pleural surface. This is particularly relevant in the context of uniportal VATS, where the reduced incision length and single working port limit both palpation and instrument triangulation.

ICG‐soaked embolisation coil placement offers a unique dual‐modality localisation benefit. The metallic coil is visible under fluoroscopy and CBCT intraoperatively and the ICG provides a strong, real‐time NIR signal during surgery, which is unaffected by surrounding blood, unlike some dyes. This allows for both radiological guidance and fluorescent visualisation.

This combination ensures that the surgeon can locate the lesion with high confidence, both at the outset of the procedure and during anatomical dissection, reducing the risk of extended resections or conversion to thoracotomy.

While many recent reports have described this technique in conjunction with advanced navigation platforms such as robotic bronchoscopy or ENB, our series demonstrates that it can be performed using only ultrathin bronchoscopy, CBCT guidance and a standard cytology brush catheter. This is a critical point for reproducibility and makes the method accessible to centres without high‐cost robotic platforms, potentially broadening its adoption across different hospital settings.

CBCT was central to the accuracy of coil placement in all four patients. The ability to perform real‐time, multiplanar reconstructions allowed precise navigation to peripheral segments, immediate confirmation of coil position and reduction of deployment errors. This is especially important when the bronchial anatomy is complex or when the lesion is in a subsegmental or angulated branch.

Additionally, Case 2 illustrates the potential of a two‐coil ‘bracketing’ strategy to address limitations of single‐coil marking in non‐subpleural lesions. By placing one coil distally at the pleural edge of the segment and another coil proximally adjacent to the lesion, the surgeon can gain a three‐dimensional sense of tumour position and segmental anatomy. This is particularly valuable when planning anatomical segmentectomy, where both lesion margins and intersegmental planes must be respected.

In Case 3, a small ICG flush was performed after coil deployment to ensure that sufficient fluorescence reached the pleural surface. This additional step can be useful when there is concern that the fluorescence intensity of the soaked coil alone may not be strong enough to be visualised intraoperatively, particularly if the coil is located deeper in the parenchyma.

Our longest localisation‐to‐resection interval was 7 days, with preserved intra‐operative fluorescence and successful R0 resection. This is consistent with prior reports showing that ICG‐soaked coils stabilise and retain a fluorescent signal that remains detectable several days after placement, thereby decoupling localisation from operating room availability [6]. In contrast, ICG‐only injections (percutaneous or bronchoscopic) are typically planned same‐day or within 24 h because diffusion and tissue turnover may attenuate signal over longer intervals [9, 10].

Although previous reports show that CT‐guided percutaneous ICG and hookwire localisation achieve high technical success with short median needle‐room times of 17 min [11], these methods carry higher complication rates, with pneumothorax, haemorrhage, and wire dislodgement being reported. Contemporary series and meta‐analyses report overall localisation‐related complications ranging roughly 30%–50%, with pneumothorax frequently the dominant event [12]. By contrast, bronchoscopic localisation (ENB or CBCT‐guided) reports show very low complication rates, as the airway approach avoids pleural puncture [13]. In our series, procedural durations ranging from 42 to 55 min were recorded and no patient experienced pneumothorax, significant bleeding, or coil migration. This mirrors the safety profile reported in other small series and case reports, suggesting that the procedure, when performed carefully, carries minimal risk.

Based on our initial experience, this technique appears most valuable for patients with small (< 15 mm), non‐palpable or non–pleural‐contact lesions. This includes cases where parenchymal preservation is a priority or when the nodule's consistency is uncertain and wedge resection may risk incomplete margins. Even when upfront anatomical resection is feasible, fluorescence‐guided coil localisation reduces uncertainty, shortens dissection time and improves segmental targeting. In our series, the mean operating time was 164 min—56 min shorter than the average operative duration prior to implementing ICG‐coil marking (220 min), where resection solely relied on visual inspection, anatomical estimation and palpation without fluorescence guidance.

From a health‐economic perspective, airway‐based localisation may reduce costs by avoiding pleural complications which can trigger additional imaging, chest tube placement, or admission. Operationally, ICG‐soaked coils enable flexible scheduling, including single‐anaesthesia pathways that combine localisation and minimally invasive resection, thereby decreasing bed utilisation and potentially enabling same‐day discharge [7]. Socially, our technique relies on widely available resources in many thoracic centres, which can shorten wait times compared with methods that require specific interventional radiology slots or specialised devices.

Although promising, further research is warranted to compare ICG coil marking with other localisation methods such as CT‐guided hookwire localisation. Despite our positive results, reliance on ICG‐soaked coils as a sole localisation strategy may have potential limitations. For deeply situated lesions, the NIR signal may not penetrate to the pleural surface, reducing detectability. Likewise, the success of the technique depends on accurate coil placement adjacent to the target, and suboptimal positioning may compromise surgical guidance. Furthermore, while coil fixation reduces diffusion and prolongs signal stability, migration or attenuation of fluorescence cannot be completely excluded. Thus, although highly effective for peripheral or subpleural nodules, its utility in central or very deep lesions remains to be fully established.

Prospective studies in larger patient cohorts could help define ideal patient selection criteria (nodule size, depth, location) as well as comparative accuracy and safety versus other localisation methods and long‐term outcomes in terms of recurrence, lung function and cost‐effectiveness.

Overall, ICG‐soaked coil placement via ultrathin bronchoscopy is a safe, reproducible and effective localisation technique, even in centres without robotic navigation systems. This series demonstrates its feasibility across diverse lesion locations and expands the applicability of fluorescence‐guided thoracic surgery to more centrally oriented nodules. The combination of ICG coil marking with uniportal VATS enabled precise, targeted resections in all cases, preserving lung parenchyma while achieving oncological R0 margins. In younger patients or those with limited pulmonary reserve, such parenchyma‐sparing strategies have long‐term functional benefits.

## Author Contributions

All the authors contributed to the manuscript. The first draft of the manuscript was written by Sammy Onyancha, and all the authors commented on previous versions of the manuscript. All the authors have read and approved the final manuscript.

## Funding

The authors have nothing to report.

## Consent

The authors declare that written informed consent was obtained for the publication of this manuscript and accompanying images using the form provided by the Journal.

## Conflicts of Interest

The authors declare no conflicts of interest.

## Data Availability

The data that support the findings of this study are available from the corresponding author upon reasonable request.
